# Brilliant green dye adsorption from aquatic medium using an activated carbon/graphene/polyacryl amide composite as a novel effective adsorbent: experimental investigation and factorial design optimization

**DOI:** 10.1039/d5ra09386d

**Published:** 2026-01-30

**Authors:** Imran Khan Rind, Muhammad Farooque Lanjwani, Ahmet Sarı, Mustafa Tuzen, Afzal Shah

**Affiliations:** a National Centre of Excellence in Analytical Chemistry, University of Sindh Jamshoro Sindh Pakistan; b Department of Human and Rehabilitation Sciences, The Begum Nusrat Bhutto Woman University Sukkur Sindh Pakistan; c Department of Metallurgical and Material Engineering, Karadeniz Technical University 61080 Trabzon Turkey; d Center of Research Excellence in Renewable Energy (CORERE), Research Institute, King Fahd University of Petroleum & Minerals (KFUPM) 31261 Saudi Arabia; e Tokat Gaziosmanpaşa University, Faculty of Science and Arts, Chemistry Department 60250 Tokat Turkey mustafa.tuzen@gop.edu.tr; f Department of Chemistry, Quaid-i-Azam University Islamabad 45320 Pakistan afzalshah@qau.edu.pk

## Abstract

The rising levels of synthetic dyes in water bodies pose significant environmental challenges due to their resistance to traditional treatment methods, chemical stability and toxicity. Recent research has focused on the development of an innovative composite material, AC/G/PAA, which combines activated carbon (AC), graphene (G) and polyacrylamide (PAA) to enhance the removal of Brilliant Green Dye (BGD). The unique properties of the functional groups in AC, combined with the characteristics of graphene and PAA, significantly enhance the adsorption capabilities of the composite. Comprehensive characterization through FTIR and SEM confirmed the successful synthesis and favorable surface properties of the AC/G/PAA composite. A Central Composite Design (CCD) model was employed to probe the influence of four independent variables on adsorption efficiency. These variables included the initial concentration, which varied from 1 to 30 mg L^−1^, contact time ranging from 10 to 50 minutes, pH levels between 3 and 8, and adsorbent dosage that spanned from 100 to 600 mg. The analysis of residual plots indicated that the model assumptions of normality, constant variance, and independence were adequately met, reinforcing the validity of the regression analysis. The equilibrium studies adhered to the Langmuir isotherm model, revealing a maximum adsorption capacity of 105.2 mg g^−1^, indicative of monolayer adsorption that exceeds many previously reported composites for BGD removal. Kinetic studies suggested that the adsorption is consistent with the pseudo-second-order kinetics, signifying a significant chemisorption mechanism. Furthermore, the AC/G/PAA composite exhibited remarkable reusability for the removal of BGD over four successive cycles, highlighting its potential as an effective, economical, and biodegradable sorbent for sustainable water treatment applications.

## Abstract 2

The rising levels of synthetic dyes in water bodies pose significant environmental challenges due to their resistance to traditional treatment methods, chemical stability and toxicity.

## Graphical abstract

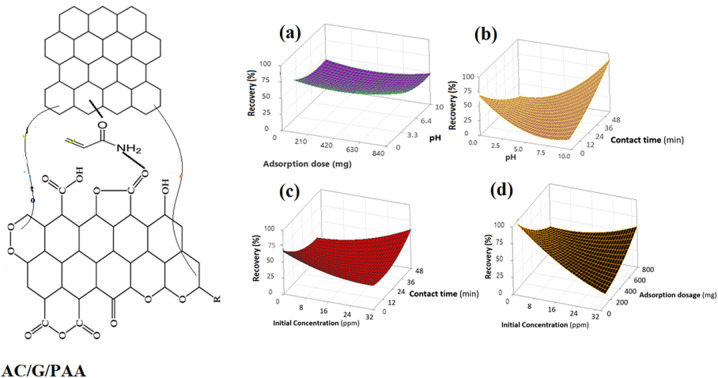

## 1. Introduction

Industrial effluents have significantly exacerbated contamination levels, adversely impacting water quality. The rapid expansion of agricultural and industrial sectors has led to the introduction of various pollutants into aquatic systems.^1^ Among these, organic dyes play a prominent role, being extensively used in industries especially in printing, leather, textiles, and paper production. The uncontrolled release and excessive application of these dyes pose serious threats to both human health and environmental sustainability. Annually, approximately 700 000 tons of dyes are produced globally, contributing to extensive pollution.^2^ One notable example is Brilliant Green Dye (BGD), which is characterized by its solid, shiny texture and deep green color, soluble in water. BGD is known to be carcinogenic, mutagenic, and toxic to living organisms, with physical contact potentially leading to severe health issues.^3^

Various techniques for removing dyes from water are currently employed, including photothermal conversion, microbial degradation, photocatalysis, electrochemical methods, and adsorption.^4^ Among these, photothermal conversion and degradation often face challenges such as limited efficiency, high energy requirements, and slow removal rates. In contrast, adsorption has emerged as a prominent technique due to the favorable chemical and physical properties of the sorbents used.^5^ This method is characterized by its high efficiency, rapid processing, and cost-effectiveness, allowing for the simultaneous capture of multiple pollutants. The adsorption process primarily occurs at the surface of the adsorbents, where they interact with contaminants. An ideal adsorbent should possess appropriate surface characteristics, a large surface area, and robust stability to facilitate the swift and effective removal of pollutants.^6^ Consequently, researchers are increasingly focusing on the synthesis and optimization of carbon-based precursor materials that are cost-effective, highly renewable, and naturally abundant.^7^

Activated carbon (AC) is a carbon-rich material distinguished by its varied pore size distribution, highly porous structure, and substantial surface area, typically around 1000 m^2^ g^−1^. It features a range of oxygenated functional groups, including phenolic, aldehyde, carboxyl, and carbonyl, which enhance its reactivity and adsorption capabilities.^8^ Additionally, AC is characterized by its low production costs, excellent chemical stability, and strong electronic conductivity. The chemical properties of AC are largely influenced by its diverse surface characteristics, which include the presence of heteroatoms such as phosphorus, sulfur, hydrogen, oxygen, and nitrogen.^9^ These attributes make AC an ideal candidate for applications in energy storage devices and the adsorption of contaminants.^10^ It can be synthesized from various natural raw materials, including fungi, bamboo, rice husk, starch, sugar, acacia gum, and other biomass sources.^11^ AC is primarily produced through two activation methods: chemical and physical, with the latter often being favored due to the absence of chemical activating agents.^12^

Graphene (G) sheets have become highly regarded for their exceptional electrical conductivity, large surface area, and impressive mechanical strength.^13^ Various methods have been employed to produce graphene layers, including epitaxial growth, micromechanical exfoliation, and chemical vapor deposition. A notable approach involves the chemical derivation of graphene sheets, where graphene oxide (GO) layers are obtained through oxidation, leading to the formation of a stable colloidal mixture due to the abundance of functional groups on their surfaces. The dispersion of nanomaterials within polymeric matrices is influenced by interfacial interactions, which can be tailored through the functional properties of the polymers and the surface chemistry of the nanomaterials.^14^ A traditional method for controlling these interactions is the surface modification of graphene sheets by adjusting the degree of oxidation.^15^ Graphene oxide sheets have been effectively used to enhance polyacrylamide (PAA), which is recognized as one of the most suitable synthetic polymeric materials for composites. This suitability arises from the strong tendency of amino groups in PAA to form hydrogen bonds with the surface functional groups of graphene, particularly in alkaline or neutral aquatic environments.^16^

Despite advancements in the development of various adsorbents, many have been hindered by issues related to environmental compatibility and insufficient adsorption capacities. While these materials exhibit potential, there is an urgent demand for sorbents that combine environmental sustainability with high efficiency and structural durability. Although progress has been made in creating carbon biopolymer based adsorbents, the need for materials that ensure strong structural integrity, high adsorption capacity, reusability, and eco-friendliness remains critical. Many of the adsorbents reported in the literature, while promising, often lack the necessary mechanical strength or only provide moderate adsorption capacities suitable for practical applications.^1,17^

The novelty of present research stems from the synergistic combination of AC, the biodegradability and hydrophobicity of graphene, and the structural stability of PAA. This integration results in a composite adsorbent that offers enhanced environmental safety, improved adsorption capacity, and better reusability. The interaction of PAA's amide groups with graphene's surface functional groups, along with the positively charged nitrogen atoms of BGD dye and the porous nature of AC significantly boosts adsorption efficiency. This novel approach seeks to overcome the limitations of conventional materials, such as costly resins and polymers, particularly regarding sustainability, economic feasibility, and performance. The study emphasizes the development of a distinctive biodegradable composite (AC/G/PAA) designed to improve environmental compatibility, structural integrity, and adsorption efficiency for the effective removal of BGD. Comprehensive experiments were meticulously designed to validate this hypothesis by examining the adsorption capabilities of the composite. The synthesized material's surface morphology and structural characteristics were analyzed using SEM and FTIR. Additionally, batch adsorption experiments were performed to optimize key variables, including pH, contact time, adsorbent dosage and solution concentration. This study seeks to present an effective, economical, and environmentally sustainable solution for the elimination of BGD from contaminated wastewater.

## 2. Materials and methods

In this research, all analytical grade chemicals were employed directly for the synthesis of the adsorbent, without any additional purification. The standard solutions were obtained from Sigma-Aldrich.

### 2.1 Synthesis of AC/G/PAA composite

The AC/G/PAA composite was synthesized following the established procedure.^18^ Graphite oxide (GO), the oxidized variant of graphite, was chemically exfoliated and reduced to form graphene sheets with thicknesses ranging from 0.8 to 1 nm. Initially, a GO solution (500 mL at mg mL^−1^ concentration) was prepared and subjected to ultrasonic exfoliation for 2 hours. Subsequently, 5 g of PAA were gradually introduced into the mixture, which was then refluxed under a nitrogen atmosphere for 24 hours at 70 °C to facilitate interaction between graphene and PAA through amine groups. Following this, 5 g AC were added and the mixture was allowed to reflux and polymerize completely for another 24 hours. The attachment of graphene and PAA to the AC was facilitated by the presence of oxygenated functional groups on their surfaces. Upon completion of the reaction, the composite was thoroughly washed with deionized water for the removal of any residual contaminants and unreacted materials. Finally, the composite was recovered from the solution through centrifugation at 10 000 rpm, resulting in the formation of the AC/PAA composite.

### 2.2 Adsorption study

The adsorption experiments were conducted using a modified adsorbent with a specific concentration of BGD in a 1 : 4 ethanol–water mixture to ensure optimal dye solubility. A representative sample of 0.02 g of the adsorbent was combined with a 10 mg per L BGD solution for various optimization processes. The separation of the adsorbent from the adsorbate was accomplished through centrifugation and filtration. The residual BGD concentration in the solution was determined using a calibration curve generated from UV-Visible spectrophotometry at the dye's maximum absorption wavelength.

The adsorption capacity (*q*_e_) and percentage efficiency (*S*%) at equilibrium for dye adsorption were evaluated using the following equations;1
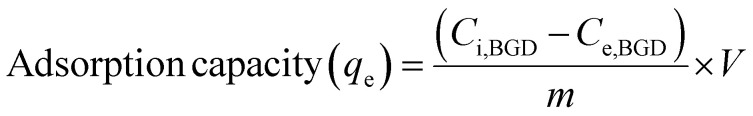
2
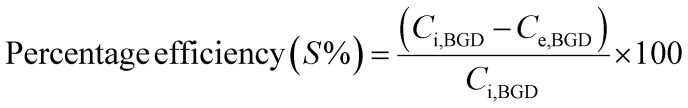
where the adsorption capacity, denoted as *q*_e_, indicates the equilibrium amount of BGD adsorbed, measured in mg g^−1^. The initial concentration of BGD is represented by *C*_i,BGD_, while *C*_e,BGD_ (mg L^−1^) refers to the equilibrium concentration. Additionally, *V* signifies the volume of the solution in L, and *m* represents the mass of the adsorbent in g.

### 2.3 Regeneration and desorption studies

In the adsorption and desorption studies, ethanol (C_2_H_5_OH) was utilized as the desorption eluent under optimal conditions as set before. After the supernatant was discarded, the AC/G/PAA composite was rinsed with distilled water. Subsequently, BGD was desorbed from the AC/G/PAA composite using 20 mL of the C_2_H_5_OH solution. The regeneration of the adsorbent was assessed by reapplying the AC/G/PAA composite following the desorption process, with the experiment conducted over ten cycles. The efficiency of BGD desorption (*R*%) was calculated using the given equation:^19^3
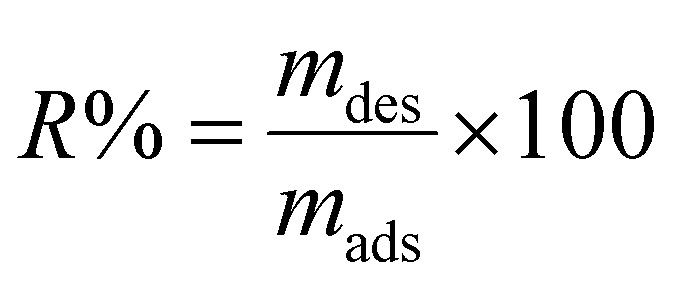
where *m*_des_ represents the desorbed amount (mg) and *m*_ads_ denoted the adsorbed amount BGD.

## 3. Results and discussions

### 3.1 Composite characterizations

SEM analysis was conducted to characterize the porous structure and surface morphology of the AC/G/PAA composite using a PHILIPS XL 30 microscope, which operates at an accelerating voltage of 20 kV and is equipped with an energy dispersive analytical system.

**Fig. 1:**
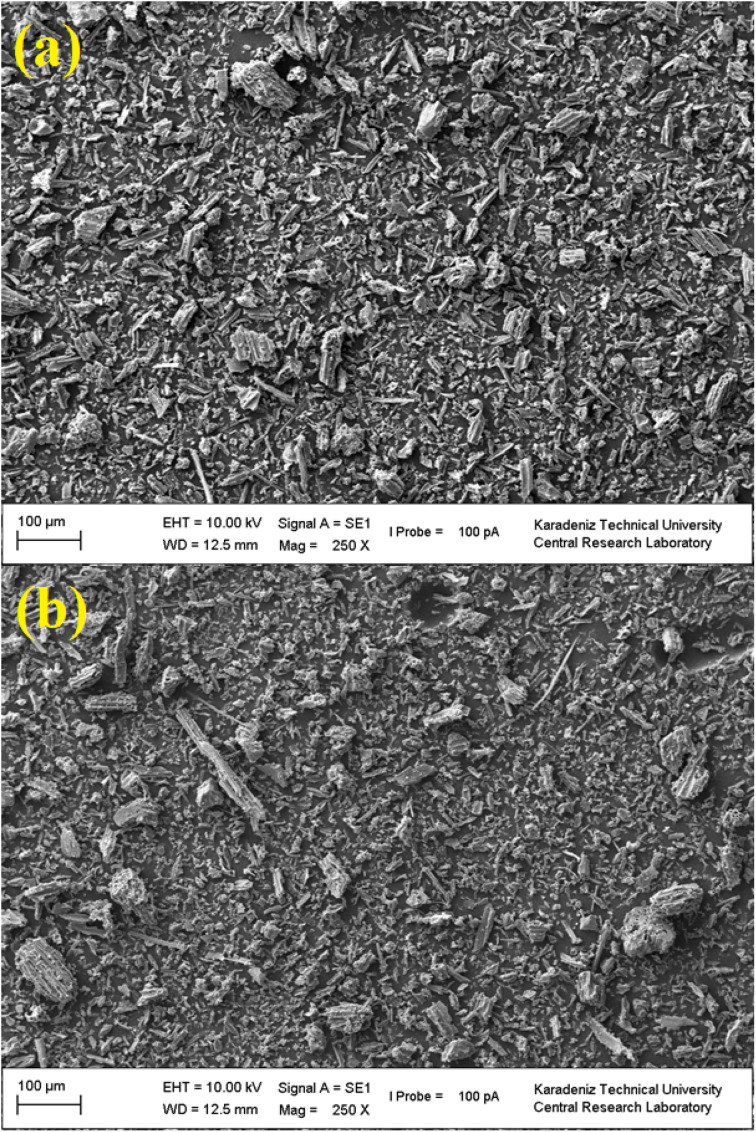
SEM micrographs of AC/G/PAA composite (a) before and (b) after BGD adsorption.

As illustrated in Fig. 1a, the AC/G/PAA structure exhibited an irregular form with rough, uneven surfaces featuring micro and macro pores of various shapes and sizes, providing effective spaces for adsorption. The incorporation of graphene allowed for the observation of wrinkles and corrugations. After the adsorption of BGD, the surface morphology of the AC/G/PAA composite exhibited signs of agglomeration, with BGD covering the pores,^20^ as illustrated in Fig. 1b.

### 3.2 Factorial design model

The Central Composite Design (CCD) model was utilized to draw the factorial design of the variables, facilitating effective optimization and interaction analysis of process parameters.^21,22^ This approach also aids in the development of robust models for process prediction and optimization. Four independent variables were examined: pH, initial concentration (*C*), adsorbent dosage (*D*), and contact time (*T*), each assessed at three coded levels: low (−), central (0), and high (+). The pH values spanned from 3 to 8, with a midpoint of 6, while the adsorbent dosage ranged from 100 mg to 600 mg, centered at 300 mg. Contact time was varied between 10 and 50 minutes, with 30 minutes as the central point, and the initial concentration of the target contaminant ranged from 1 ppm to 30 ppm, with a central value of 10 ppm (Table 1). These defined ranges enable the evaluation of both linear and quadratic effects of each factor. A total of 21 experimental runs were conducted to optimize the process variables using the CCD approach, with recovery percentages fluctuating significantly from 22% in experiment 13 to 93% in experiment 21 (Table 2). Notably, experiments conducted at the central point consistently yielded the highest recovery rates, suggesting an optimal region around these central values. This structured design allows for a comprehensive assessment of both interactive and individual effects of the variables on recovery efficiency.

**Table 1:** Factorial design of variables using CCD model

Variable	Low (−)	Central point (0)	High (+)
pH	3	6	8
Adsorbent dosage (*D*) (mg)	100	300	600
Contact time (*T*) (min)	10	30	50
Initial concentration (*C*) (ppm)	1	10	30

**Table 2:** Experimental run for optimization of variables

Experiment	pH	*D*	*T*	*C*	Recovery(%)
1	+	+	+	+	50
2	+	+	+	−	62
3	+	+	−	+	58
4	+	+	−	−	49
5	+	−	+	+	32
6	+	−	+	−	54
7	+	−	−	+	45
8	+	−	−	−	40
9	−	+	+	+	30
10	−	+	+	−	45
11	−	+	−	+	40
12	−	+	−	−	57
13	−	−	+	+	22
14	−	−	+	−	40
15	−	−	−	+	24
16	−	−	−	−	37
17	0	0	0	0	86
18	0	0	0	0	88
19	0	0	0	0	90
20	0	0	0	0	91
21	0	0	0	0	93

#### 3.2.1 Variance analysis (ANOVA)

ANOVA is a statistical technique used to assess significant differences in means among different groups or variables.^23^ This analysis utilizes a model to optimize the effects of different variables on recovery, as illustrated in Table 3. The overall model yielded a *p*-value of 0.062, which is marginally non-significant, suggesting a moderate fit for the data. Among the linear factors, both adsorbent dosage (*p* = 0.043) and initial concentration (*p* = 0.031) significantly influenced recovery, while pH and contact time did not show individual significance. The squared terms were highly significant (*p* = 0.017), highlighting the importance of curvature in the model, with all four squared terms (pH, dosage, time, concentration) demonstrating strong effects (*p* < 0.05), indicating non-linear relationships. Furthermore, significant interaction was noted in the two-way interaction terms (*p* = 0.039), particularly between pH and concentration, which exhibited a statistically significant interaction (*p* = 0.035), reflecting their combined effect on recovery. Other interaction terms did not achieve significance. The lack-of-fit test was not significant (*p* = 0.514), suggesting that the model fits the data well without considerable unexplained variation.

**Table 3:** Variance analysis (ANOVA)

Source	*F*-Value	*p*-Value
Model	3.61	0.062
Linear	2.51	0.150
pH	0.01	0.918
Adsorbent dosage (*D*) (mg)	25.79	0.043
Contact time (*T*) (min)	0.57	0.479
Initial concentration (*C*) (ppm)	17.88	0.031
Square	27.43	0.017
pH × pH	21.46	0.023
Adsorbent dosage (*D*) (mg) × adsorbent dosage (*D*) (mg)	33.16	0.026
Contact time (*T*) (min) × contact time (*T*) (min)	32.06	0.022
Initial concentration (*C*) (ppm) × initial concentration (*C*) (ppm)	21.17	0.004
2-Way interaction	31.84	0.039
pH × adsorbent dosage (*D*) (mg)	0.19	0.681
pH × contact time (*T*) (min)	0.03	0.874
pH × initial concentration (*C*) (ppm)	25.76	0.035
Adsorbent dosage (*D*) (mg) × contact time (*T*) (min)	0.27	0.623
Adsorbent dosage (*D*) (mg) × initial concentration (*C*) (ppm)	0.98	0.361
Contact time (*T*) (min) × initial concentration (*C*) (ppm)	0.49	0.509
Lack-of-Fit	0.49	0.514

#### 3.2.2 Residual graphs

**Fig. 2:**
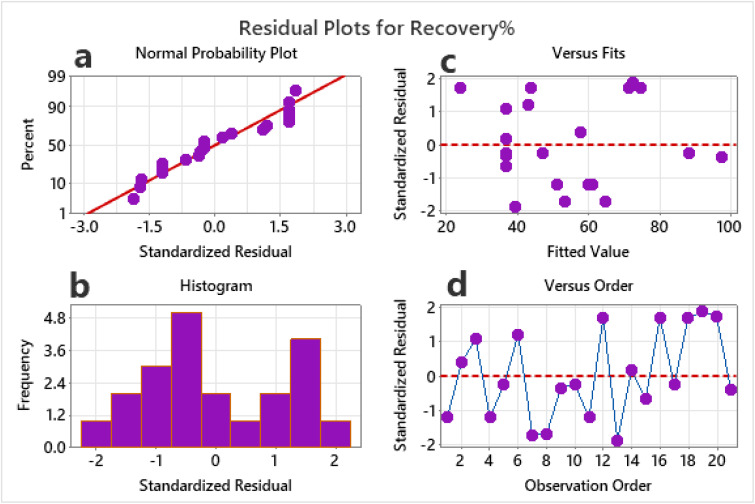
Residual plots: (a) standardized residual *versus* percent, (b) standardized residual *versus* frequency, (c) fitted value *versus* standardized residual, and (d) observation order *versus* standardized residual for recovery of BGD using the AC/PAA composite adsorbent.

Residual plots are diagnostic graphs that display the errors, or residuals, of a regression model. They are used to examine the key assumptions of linear regression, such as linearity and constant variance. Ideally, the points should be randomly scattered around zero with no discernible patterns. Any systematic patterns, like curves or funnels, indicate a problem with the model fit. Analyzing these plots is essential for validating and improving the model's accuracy.^24^ The residual plots used for optimization of recovery% of BG dye, which are essential for diagnosing the adequacy of the regression model and its assumptions. Normal probability plot (Fig. 2a): the standardized residuals mostly follow the straight red line, suggested that the distribution of residuals is close to normal. This supports the normality assumption in the error terms. Histogram (Fig. 2b): the histogram of standardized residuals appears roughly symmetrical and bell-shaped, which further confirms the normality assumption, though it shows some mild skewness. Residuals *vs.* fitted values (Fig. 2c): residuals are distributed randomly around zero with no clear pattern, indicating that variance is constant (homoscedasticity). However, some clustering near higher fitted values could hint at minor non-linearity or influential points. Residuals *vs.* observation order (Fig. 2d): the residuals exhibit random fluctuations without any evident trend. This suggests that there is no autocorrelation or time-related bias in the data collection process. Overall, the residual plots indicate that the model assumptions of normality, constant variance, and independence are reasonably satisfied, supporting the reliability of the regression analysis.

#### 3.2.3 Surface plots

**Fig. 3:**
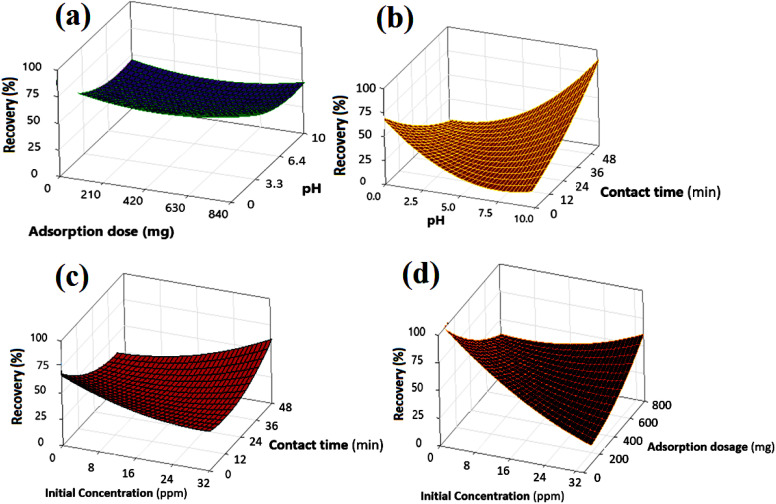
Response surface plot of recovery% between (a) adsorption dosage and pH (b) pH and contact time, (c) contact time and initial concentration and (d) adsorption dosage and initial concentration.

Response surface plots (3D plots) are graphical representations used in Response Surface Methodology (RSM) to visualize how changes in two independent variables simultaneously affect a dependent variable (response), while keeping other variables constant.^25,26^Fig. 3a represents the response plot of recovery (%) *vs.* pH and adsorption dosage. The surface plot investigates the interplay between the solution's pH and the adsorption dosage. It reveals that pH is a crucial parameter for optimizing the process. The plot shows that the highest recovery (again in red) is not achieved at the extremes of the pH range but within a specific, moderately acidic window, approximately between pH 3.3 and 6.4, combined with a sufficiently high adsorption dose. This optimal pH range is likely where adsorbent surface charge and ionic form of the solute create the strongest electrostatic attraction or chemical affinity for adsorption. At very low pH (highly acidic) or higher pH (approaching neutral), the recovery decreases for a given adsorbent dose, as shown by the green and blue regions. This indicates that the adsorption mechanism is highly pH-sensitive. The Fig. 3b is a 3D plot illustrating the relationship between pH and contact time, *vs.* recovery. Plot's *x*-axis shows pH, varied from 0.0 to 10.0, while *y*-axis shows contact time in minutes, from 0 to 48. Recovery percentage is shown through a color gradient and contour lines, with red indicating the highest recovery (near 100%) and blue the lowest. A distinct optimal region for maximum recovery is visible in the upper-left section of the plot. This region corresponds to a combination of low pH (highly acidic conditions, approximately 0.0 to 2.5) and long contact times (approximately 36 to 48 minutes). As the pH increases beyond this highly acidic range, the recovery percentage decreases significantly, even with extended contact times. Similarly, at any given pH, shorter contact times consistently result in lower recovery. Fig. 3c showed the recovery (%) *vs.* contact time and initial concentration.

The dimensional plots illustrate the direct influence of the initial solute concentration on the recovery percentage, revealing a strong inverse relationship. As the initial concentration increases, the recovery percentage declines sharply. Beyond a certain concentration threshold, recovery efficiency plummets, stabilizing at approximately 25–30% for higher concentrations (24–30 ppm). This behavior is characteristic of adsorption processes, highlighting the finite capacity of the adsorbent material. At lower concentrations, there are sufficient active sites available on the adsorbent to effectively capture solute molecules. However, as concentration rises, these sites become saturated, resulting in a significant amount of solute remaining in the solution and consequently reducing the overall recovery percentage. The contour plot in Fig. 3d illustrates the recovery percentage in relation to both adsorbent dosage and initial concentration, with a color gradient and surface lines indicating a high-performance zone. The optimal recovery (depicted in red, exceeding 90%) occurs in areas of low initial concentration coupled with high adsorption dosage, which is logical since a larger quantity of adsorbent can efficiently manage a dilute solution. Conversely, as one move towards higher concentrations (rightward) or lower dosages (downward), the recovery drops, transitioning through yellow and green to blue. This visualization powerfully demonstrates the trade-off between these two factors. To maintain a high recovery rate when treating more concentrated solutions, one must significantly increase the amount of adsorbent used.

#### 3.2.4 Pareto chart

**Fig. 4:**
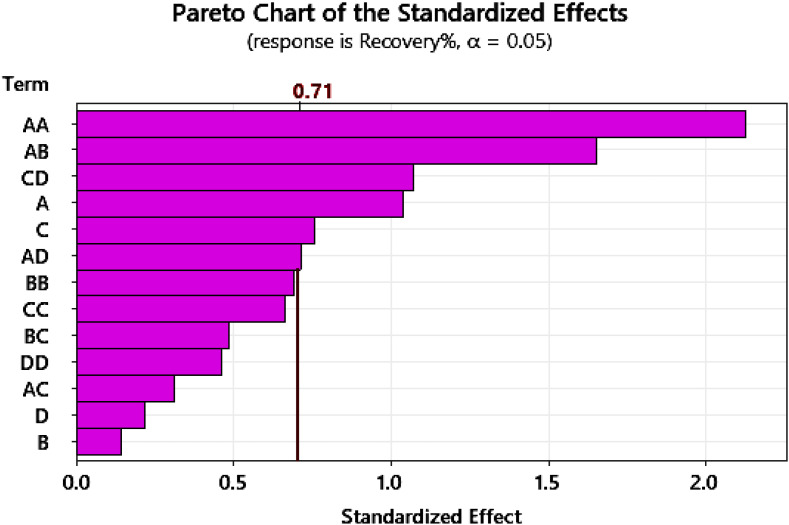
Pareto chart of variables.

A Pareto chart is a specialized bar graph that combines both bars and a line to prioritize factors. The bars represent individual values (like frequency or cost) in descending order from left to right. This visual arrangement instantly highlights the most significant items.^27^ A cumulative line is overlaid on the bars, showing the total percentage contributed as you move to the right. The core principle behind it is the principle of Pareto, often called the 80/20 rule. This rule suggested roughly 80% of the effects came from 20% of the causes. Based on the provided Pareto chart of standardized effects for the response “Recovery%”, the following observations can be made. The chart identifies which process factors and their interactions have a statistically significant effect, using an alpha level of 0.05 (Fig. 4). In Pareto chart A denotes pH, B the adsorbent dosage, C the contact time and D the initial concentration. The two-factor interactions AA and AB, along with the interaction CD, are the most dominant effects, as their bars clearly cross the significance threshold. The main factors A and C also show a substantial influence on the recovery percentage. Conversely, several other terms exhibit a lesser impact. The interactions AD, BB, and CC, as well as BC and DD, fall below the significance line, indicating their effects are not statistically strong. The remaining terms, including the AC interaction and the main factors B and D, demonstrate the smallest standardized effects and are therefore considered the least influential on the process outcome. This chart successfully prioritizes the key variables, suggesting that optimization efforts should focus primarily on factors A and C and their interactions.

### 3.3 Adsorption equilibrium study

#### 3.3.1 Isotherm models

In the adsorption process, contaminants attach to the solid material surface, reaching equilibrium when the concentration of water stabilizes alongside the amount of contaminants that have been adsorbed. The adsorption isotherm effectively depicts the correlation between the quantities of contaminants that are adsorbed and their concentrations in the water, thereby clarifying the nature of this equilibrium state.^28^ Various adsorption parameters can be calculated, with the Langmuir and Freundlich isotherms successfully representing the equilibrium adsorptive behavior observed in the removal of BGD. The Langmuir isotherm characterizes a uniform adsorption process occurring at the active sites of the adsorbent, where adsorption ceases once these sites are fully occupied, indicating a monolayer formation. In contrast, the Freundlich isotherm serves as an empirical model that accommodates multilayer adsorption on a heterogeneous adsorbent surface.^29^

Langmuir and Freundlich non-linear isotherms equations are given as:4
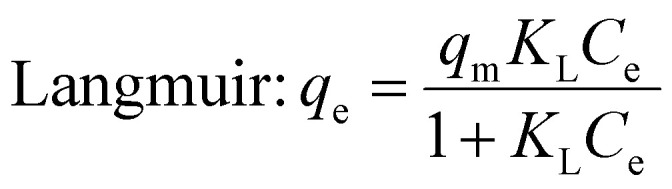
5
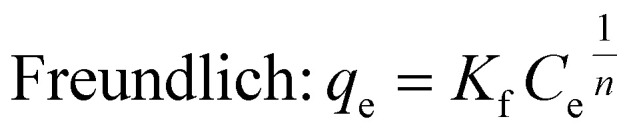
where *q*_e_ represents the equilibrium amount of BGD adsorbed (mg g^−1^), while *C*_e_ denotes the equilibrium concentration of BGD (mg L^−1^). The Langmuir constants, *K*_L_ and *q*_m_, correspond to the equilibrium constant (L mg^−1^) and the maximum adsorption capacity (mg g^−1^), respectively. In the Freundlich model, the parameter *n* indicates the sorption intensity and reflects the bond energy between the dye and the composite, whereas *K*_f_ signifies the Freundlich constant, which is associated with multilayer adsorption and the overall adsorption capacity.

**Fig. 5:**
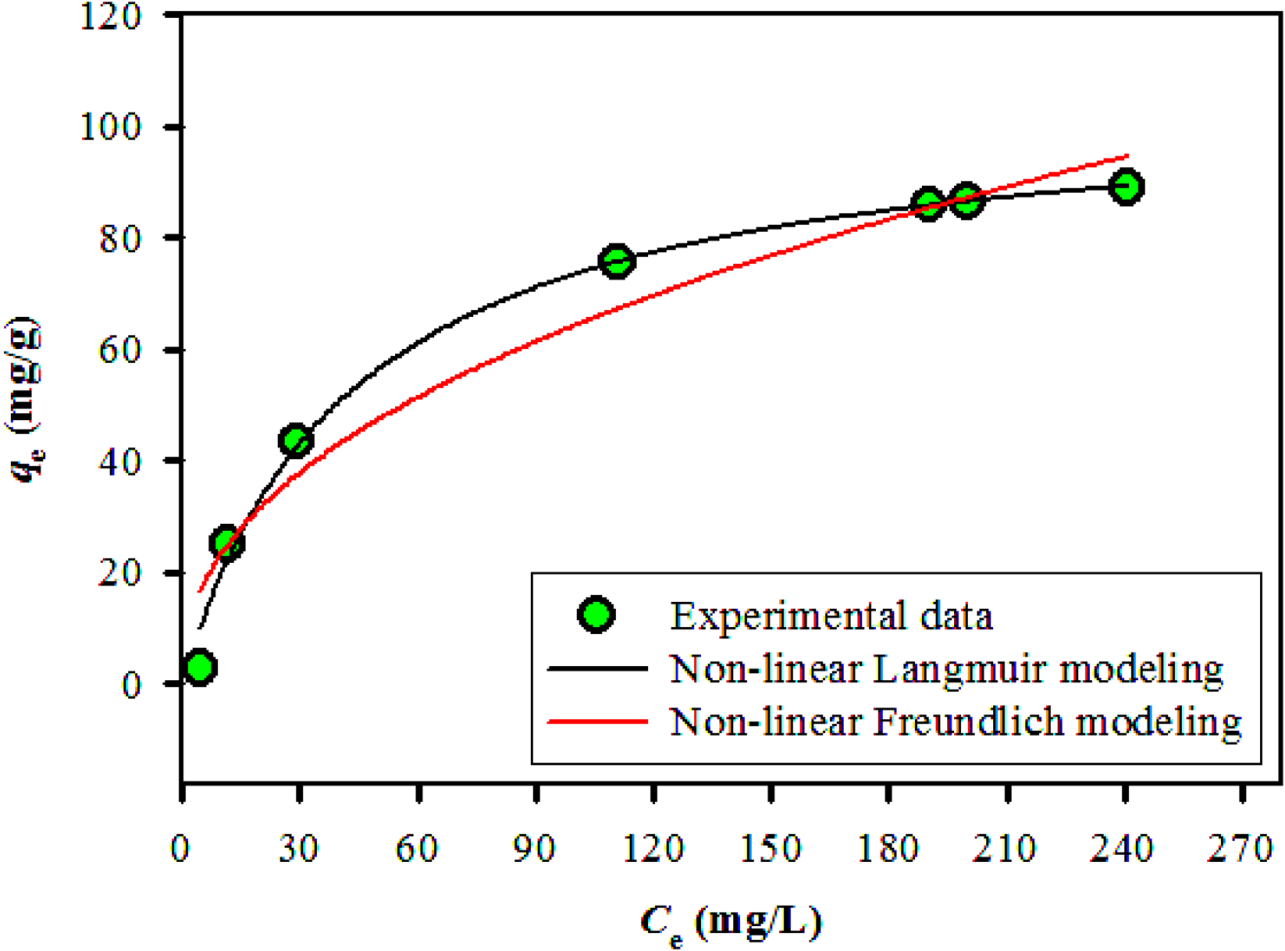
The isotherm modeling results for BGD removal by AC/G/PAA at 24 °C.

The Langmuir and Freundlich non-linear regression isotherm models were utilized to analyze the experimental equilibrium data regarding the adsorption of BGD onto the AC/G/PAA composite adsorbent. The adsorption data for these isotherm models is illustrated in Fig. 5, while Table 4 presents the parameter values and constants for both the Langmuir and Freundlich isotherms. This analysis highlights the variation in the equilibrium adsorbed amount in relation to the initial concentrations of the solutions.

**Table 4:** Isotherm models parameters for the removal of BGD using AC/G/PAA composite (pH: 3, adsorbent dosage: 100 mg L^−1^; contact time: 30 min; temperature: 24 °C)

Non-linear isotherm modeling data	Parameter	Value
Langmuir	*q*_m_ (mg g^−1^)	105.2
	Std error	0.29–3.5 × 10^−3^
	*R* ^2^	0.992
	*K*_L_	2.33 × 10^−2^
Freundlich	*K*_f_ (mg g^−1^)	8.54
	Std error	2.92–6.68 × 10^−2^
	1/*n*	0.439
	*R* ^2^	0.954

The results from the Langmuir isotherm indicate a strong correlation, with an *R*^2^ value of 0.992 and a relatively low standard error range of 0.29 to 3.5 × 10^−3^. In contrast, the Freundlich model exhibited relatively higher standard error range (2.92 to 6.68 × 10^−2^) and a lower regression correlation coefficient of *R*^2^ = 0.954. The Langmuir isotherm implies a monolayer adsorption mechanism, effectively fitting the data related to the removal of BGD using the AC/G/PAA composite, which demonstrated a maximum adsorption capacity of 105.2 mg g^−1^. The adsorption process on the surfaces of the AC/G/PAA composite occurs uniformly, suggesting that the adsorption of BGD takes place with consistent energy across fewer adsorption sites.

#### 3.3.2 Adsorption kinetics

The kinetic adsorption mechanism elucidates the process of pollutant adsorption by analyzing key parameters related to sorption kinetics. Two primary models have been proposed: pseudo first order (PFO) and pseudo second order (PSO) kinetics. The PFO model is a straightforward approach commonly applied to sorption processes in solid/liquid systems, while the PSO model is based on the premise that the sorption process adheres to second order chemisorption dynamics.^2^

PFO and PSO non-linear kinetic models equations are represented as:

6 PFO: *q*_*t*_ = *q*_e_(1 − e^−*k*_1_*t*^)

7
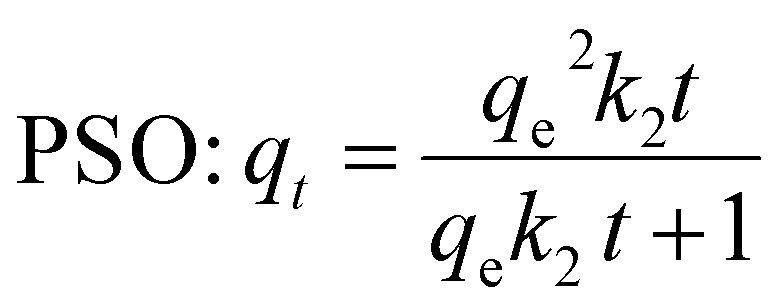
where *k*_1_ is the PFO BGD adsorption rate constant (min^−1^), *q*_e_ and *q*_*t*_ are the BGD adsorbed amounts (mg g^−1^) at equilibrium and at time *t* (min). While *k*_2_ (g mg^−1^ min^−1^) refers to PSO kinetic adsorption rate constant.

**Fig. 6:**
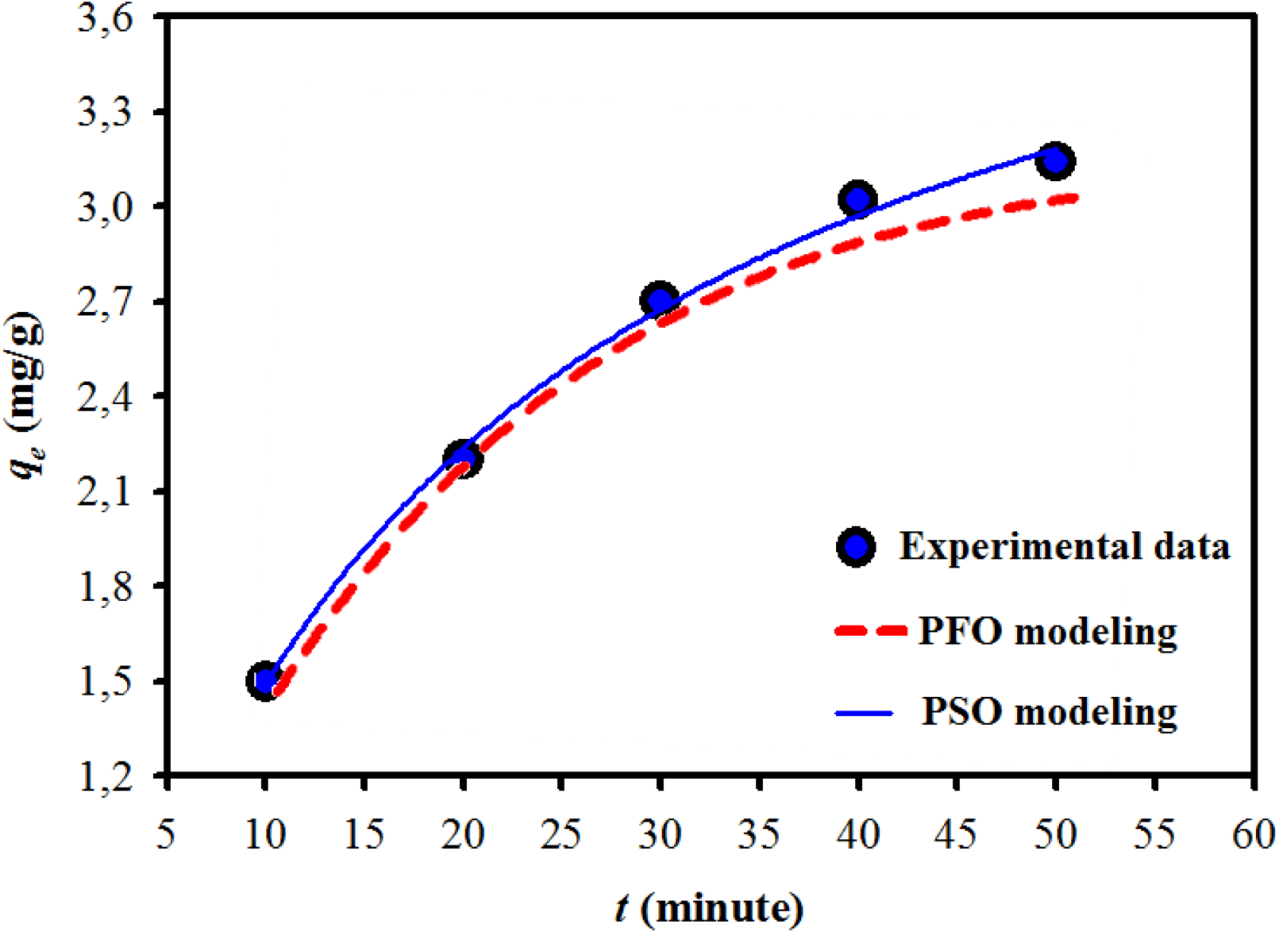
Kinetic results obtained for BGD adsorption using AC/G/PAA composite at 24 °C.

Fig. 6 illustrates the parameters of the PFO and PSO kinetic models derived from non-linear plots. These adsorption kinetic models assess the interactions between BGD and the AC/G/PAA composite, focusing on the rate-controlling steps that include material transport and physical–chemical reactions. Table 5 presents the rate constants (*k*_1_, *k*_2_) and the correlation coefficients (*R*^2^) for both models. The findings indicate a significant discrepancy between the calculated equilibrium uptake capacity (*q*_e1,cal_ = 1.39 mg g^−1^) and the experimental equilibrium uptake capacity (*q*_e,exp_ = 2.92 mg g^−1^), with the PFO model showing a lower correlation coefficient (*R*^2^ = 0.969) and with relatively higher standard error range (4.18 to 8.23 × 10^−2^). In contrast, the kinetic adsorption data align closely with the PSO model, as demonstrated by a much higher correlation coefficient (*R*^2^ = 0.997) and with relatively lower standard error range of 0.36 to 2.82 × 10^−3^. Moreover, a minimal difference between the calculated value (*q*_e2,cal_ = 2.78 mg g^−1^) and the experimental value (*q*_e,exp_ = 2.92 mg g^−1^). This kinetic study highlights the effective adsorption of BGD onto the AC/G/PAA composite, attributed to the availability of binding sites. The chemisorption process involves a rate-controlling step related to electron sharing for chelate formation, as well as interactions between the AC/G/PAA composite and BGD through valence forces and electron exchange.

**Table 5:** Adsorption kinetic results using AC/G/PAA composite for BGD removal at 24 °C (pH: 3, adsorbent dosage: 100 mg L^−1^; contact time: 30 min; temperature: 24 °C)

Kinetic models	Parameters	AC/G/PAA
PFO	*q*_e,exp_ (mg g^−1^)	2.92
	*k*_1_ (min^−1^)	4.36 × 10^−3^
	*q*_e1,cal_ (mg g^−1^)	1.39
	*R* ^2^	0.969
PSO	*k*_2_ (g mg^−1^ min^−1^)	1.42 × 10^−4^
	*q*_e2,cal_ (mg g^−1^)	2.78
	*R* ^2^	0.997

### 3.4 Thermodynamics results for BGD adsorption by AC/G/PAA

Thermodynamic behavior of BGD adsorption by AC/G/PAA was investigated at temperature of 297, 307, 317, 327 and 337 K. The *K*_e_ equilibrium constants calculated based on these temperatures was used for calculation of Gibbs free energy (Δ*G*) function as given below:8
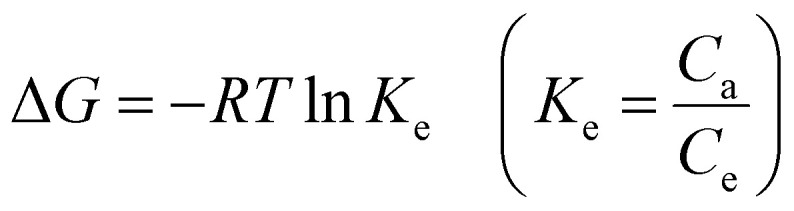
where, *C*_a_ and *C*_e_ symbolize the adsorbed concentration and the equilibrium concentration of BGD (mg L^−1^). Also, the enthalpy (Δ*H*) and entropy (Δ*S*) functions were calculated based on the following equation.9
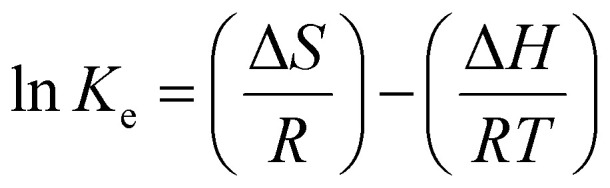


**Fig. 7:**
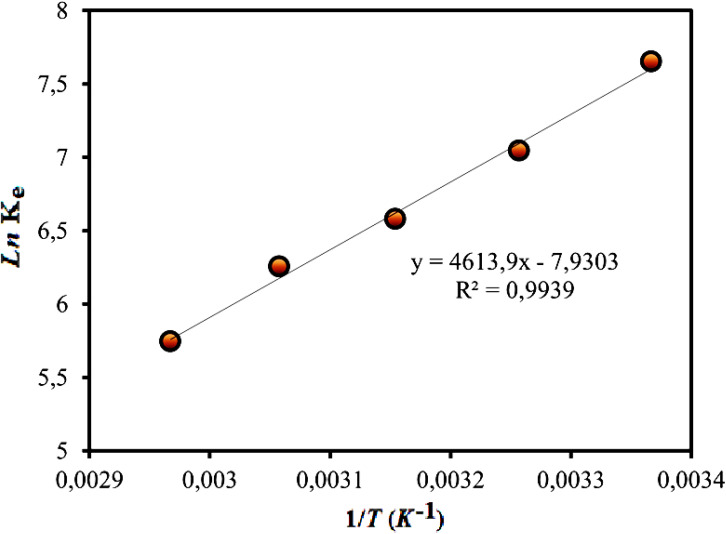
Change of *K*_e_ depending on 1/*T*.

The change in *K*_e_ value was plotted *versus* 1/*T* values as shown in Fig. 7. The Δ*G* function was calculated as −18.8, −17.9, −17.3, −17.0 and −16.1 kJ mol^−1^ for 297, 307, 317, 327 and 337 K, respectively. These data suggested that the BGD adsorption onto AC/G/PAA composite was occurred spontaneously. By considering the data given in Fig. 7, the Δ*H* function (−38.4 kJ mol^−1^) verified the exothermic and physisorption nature of the adsorption process. The Δ*S* function (−65.9 J mol^−1^ K^−1^) revealing the declined complication at the solution–adsorbent interface for the considered temperatures. Similar thermodynamic results have also been reported for the adsorption of methylene blue dye using biomass derived-activated carbon.^29^

### 3.5 Regeneration

**Fig. 8:**
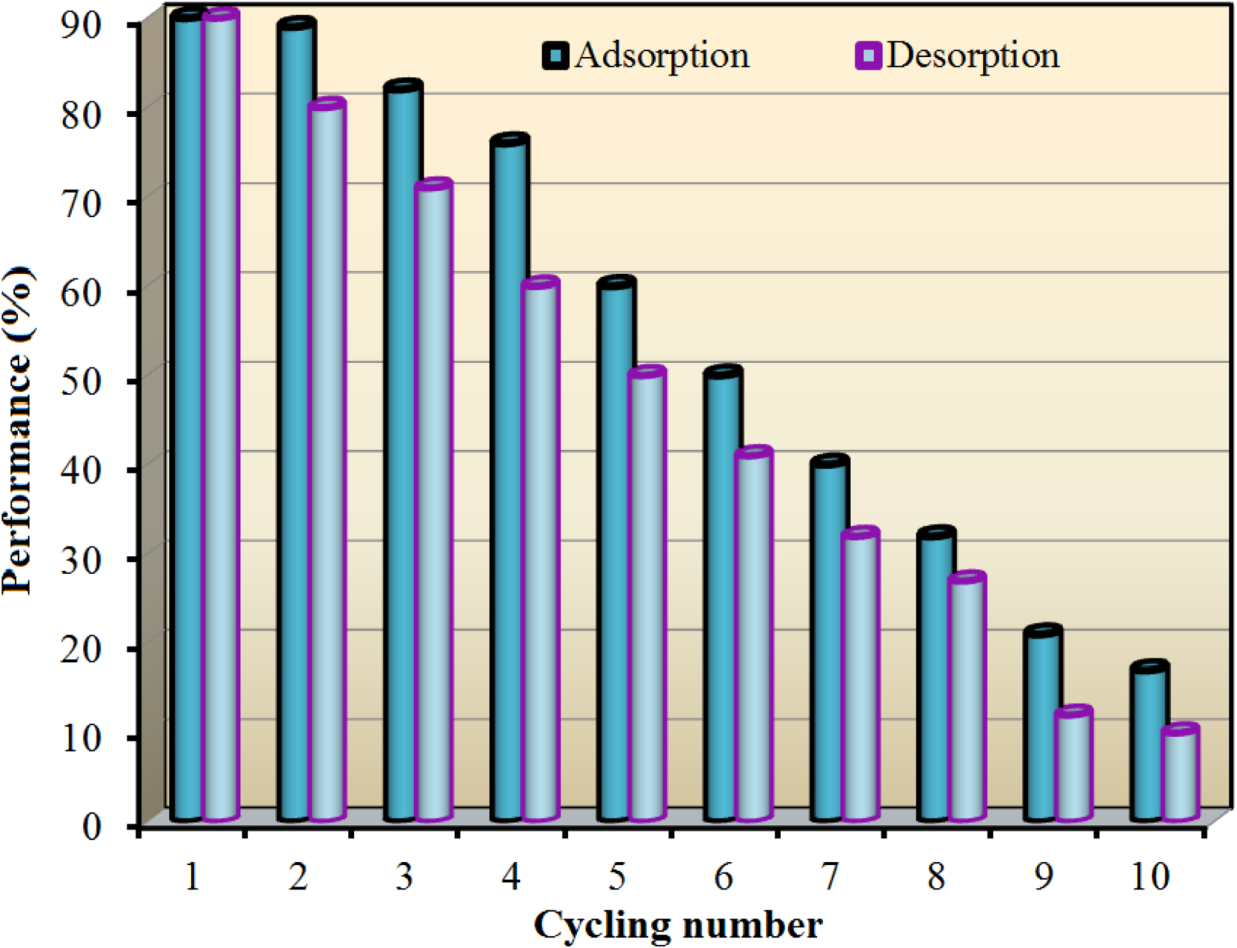
Cycling reuse performance for BGD adsorption using AC/G/PAA composite at 24 °C.

The AC/G/PAA composite underwent ten recycling cycles in air following regeneration. The results regarding the recyclability of AC/G/PAA and the adsorption isotherm across various cycles are illustrated in Fig. 8. The adsorption–desorption yields for the first, third, fifth, and tenth cycles were recorded at 90/90%, 82/71%, 60/50%, and 17/10%, respectively. These results indicate that the AC/G/PAA composite exhibits significantly higher BGD adsorption, highlighting the crucial role of its porous structure and composite nature in enhancing adsorption capacity. Based on these observations, the AC/G/PAA composite demonstrates effective adsorption capabilities at 24 °C for up to five cycles.

### 3.6 The comparison study

The performance of AC/G/PAA composite to adsorb BGD is notably superior compared to other similar composite-based adsorbents reported recently, as summarized in Table 6. This suggests that the porous architecture of the AC/G/PAA composite positions it as a highly effective material for the removal of various pollutants from aquatic environments.

**Table 6:** Comparison of the adsorption capacities of AC/G/PAA against several other adsorbents used for BGD adsorption

Adsorbent	Adsorption capacity (mg g^−1^)	pH	Temperature (K)	Reference
Modified areca nut husk	18.2	7.0	298	Ref. 30, https://doi.org/10.1016/j.sajce.2020.11.001
Nutraceutical industrial pepper seed spent	223.6	7.0	323	31
AC derived from guava seeds	80.5	9.0	298	Ref. 32, https://www.deswater.com/DWT_articles/vol_202_papers/202_2020_396.pdf
Mercerized pistachio shell	52.42	—	300	Ref. 33, https://www.mdpi.com/1420-3049/28/10/4129
Peanut shells	19.9	9.0	303	Ref. 34, https://www.mdpi.com/1420-3049/28/10/4129
Watermelon peels	25.0	8.0	303	Ref. 35, https://sustainability.uobabylon.edu.iq/uploaded/Sustain_2022_101053584.pdf
Rice straw	30.7	—	293	Ref. 36, https://journalajacr.com/index.php/AJACR/article/view/4
Sawdust from Indian *Eucalyptus* wood	58.5	2.9	288	Ref. 37, https://www.sciencedirect.com/science/article/pii/S0011916411000609?via%3Dihub
Red clay	125.0	7.0	318	Ref. 38, https://www.sciencedirect.com/science/article/pii/S1385894713005925?via%3Dihub
Used tea powder	101.0	6.0	308	Ref. 39, https://iwaponline.com/aqua/article/71/10/1148/91093/Adsorption-of-brilliant-green-dye-by-used-tea
Nano hydroxyapatite/chitosan composite	49.1	7.0	323	Ref. 40, https://www.mdpi.com/1420-3049/24/5/847
AC	51.8	6.0	297	This work
AC/G/PAA	105.2	6.0	297	This work

### 3.7 Adsorption reaction mechanism

**Fig. 9:**
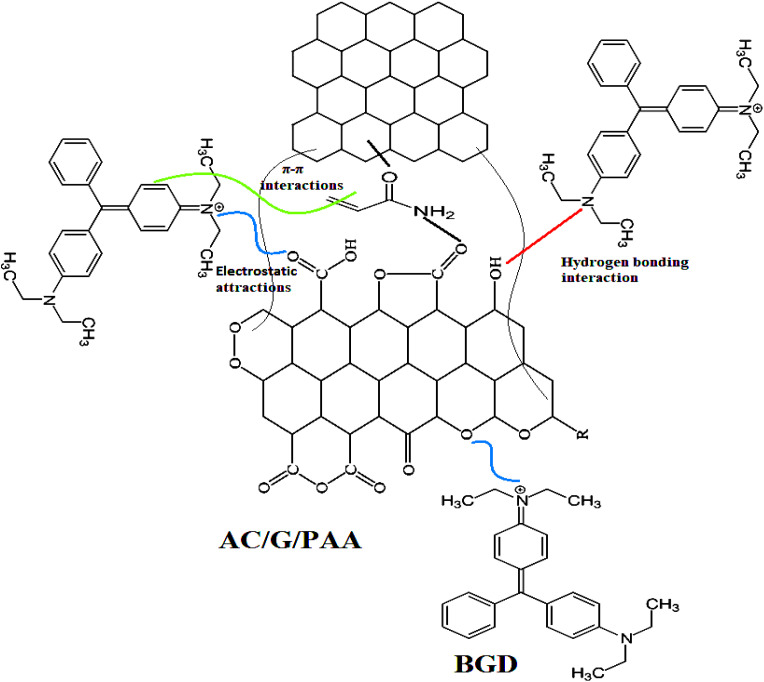
Proposed adsorption mechanism between AC/G/PAA composite and BGD.

The chemical structure and interaction mechanism of the BGD with the AC/G/PAA composite are illustrated in Fig. 9. The adsorption reaction mechanism is influenced by various factors, including the physiochemical and structural properties of the adsorbent. Additionally, the type of interaction between the adsorbate and adsorbent plays a crucial role in the effectiveness of contaminant removal.

**Fig. 10:**
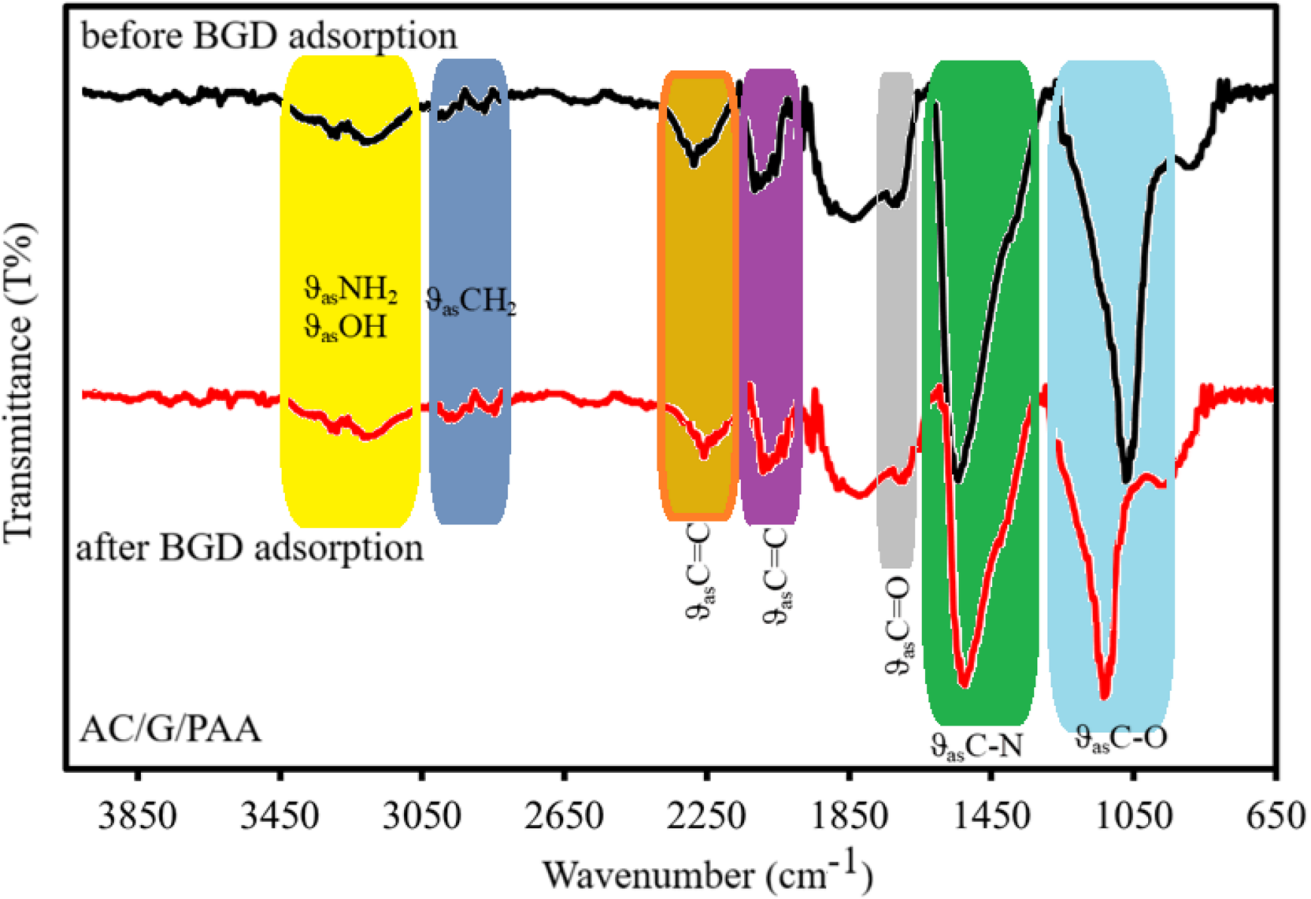
FT-IR spectra of AC/G/PAA composite before and after BGD removal.

In this study, we have demonstrated the adsorption mechanism by comparing the results of FTIR spectroscopy conducted before and after the adsorption of Brilliant Green Dye onto the AC/G/PAA composite adsorbent as illustrated in Fig. 10. The spectral analysis of the composite prior to adsorption reveals several significant peaks. The peaks observed at 3251 and 3260 cm^−1^ correspond to the asymmetric stretching (*θ*_as_) bands of –N–H or O–H groups present in the structures of PAA or AC. Additionally, the bands at 2906 and 2998 cm^−1^ indicate the asymmetric stretching of –CH_2_/C–H groups associated with PAA or AC. A peak at 1742 cm^−1^ is linked to the C=O group of PAA or AC, while the peaks at 2292 and 2119 cm^−1^ pertain to the asymmetric stretching of C=C groups found in AC, graphene, or PAA. Furthermore, the stretching peak at 1555 cm^−1^ is attributed to the asymmetric stretching of the C–N group in PAA, and the peak at 1077 cm^−1^ is associated with the C–O groups of AC or graphene.

A comparison of the spectral results obtained after the adsorption process revealed notable shifts in the characteristic bands of the composite components. Specifically, the wavenumbers for the asymmetric stretching bands of –N–H and O–H groups in PAA or AC shifted to 3254 and 3263 cm^−1^, respectively, while the asymmetric stretching bands of CH_2_/C–H groups changed to 2901 and 2994 cm^−1^. Additionally, the C=C stretching band associated with AC or graphene shifted to 2286 and 2116 cm^−1^, and the C–N stretching band varied to 1542 cm^−1^. The C=O groups exhibited a peak at 1738 cm^−1^, with the C–O group peaks recorded at 1042 cm^−1^. These wavenumber shifts for the N–H, O–H, C=O, and C–O groups in PAA or AC can be attributed to the formation of hydrogen bonds and electrostatic attractions with the positively charged nitrogen atoms of BGD molecules during adsorption. Furthermore, the changes in the stretching wavenumber of C=C groups in AC or graphene may result from π–π interactions with the benzene rings present in BGD molecules. Similar observations regarding the adsorption mechanisms between various dye molecules and the surface functional groups of different adsorbents have been documented in the literature.^41–45^

## 4. Conclusions

Increasing synthetic dyes discharge into aqueous solutions has become a primary environmental concern due to their resistance to conventional treatment methods, high chemical stability and toxic nature. This study introduces a novel biodegradable composite made from AC, graphene, and PAA, creating a multifunctional material designed for enhanced dye removal. By combining the porosity of AC with the structural integrity of PAA and the diverse functionalities of graphene, the resulting hybrid material demonstrates exceptional performance in removing BGD. SEM images reveal a porous network structure within the AC/G/PAA composite. Response Surface Methodology visualized changes in two independent variables simultaneously affecting a dependent variable (response). The adsorption kinetics aligned well with the Pseudo-Second Order model, while the BGD adsorption followed the Langmuir isotherm, achieving an impressive capacity of 105.2 mg g^−1^, indicative of monolayer adsorption primarily driven by chemisorption. Furthermore, reusability tests confirmed the composite's stability and effective removal efficiency over four cycles, highlighting its potential as a reliable sorbent for repeated applications. The driving forces that facilitated BGD removal were π–π interactions, electrostatic attractions, and hydrogen bonding. Results established the AC/G/PAA composite as an effective and environmentally friendly agent for purifying water contaminated with toxic dyes.

## Conflicts of interest

There are no conflicts of interest to declare.

## Data Availability

The authors declare that the data are available in this manuscript in the form of tables and figures.
